# Analysis of the composition of culturable airborne microorganisms in the archaeological excavation protection site of the Nanhai No. 1 Ancient Shipwreck

**DOI:** 10.3389/fmicb.2022.958914

**Published:** 2022-08-25

**Authors:** Kaixuan Ma, Yu Wang, Xinyu Yang, Cen Wang, Yeqing Han, Xinduo Huang, Peifeng Guo, Jing Du, Yue Chen, Jiao Pan

**Affiliations:** ^1^Key Laboratory of Molecular Microbiology and Technology of the Ministry of Education, Department of Microbiology, College of Life Sciences, Nankai University, Tianjin, China; ^2^National Center for Archaeology, Beijing, China; ^3^Institute for Cultural Heritage and History of Science and Technology, University of Science and Technology Beijing, Beijing, China

**Keywords:** Nanhai No. 1 Ancient Shipwreck, culturable airborne microorganisms, biocorrosion, biodegradation, potential harmful microorganisms

## Abstract

After the recovery of the ship from the sea on 2007, the Nanhai No. 1 Ancient Shipwreck is currently exposed to the air. Air microorganisms settle on wooden shipwrecks, and they can use wood matrix to grow and multiply, causing biocorrosion and biodegradation. In this study, a systematical survey of the composition of culturable airborne microorganisms was performed at the conservation site of the Nanhai No. 1 Ancient Shipwreck. Airborne microorganisms were collected from seven sites in the preservation Nanhai No. 1 area over five periods. Molecular identification of the culturable microorganisms isolated from the air was done by sequencing both 16S rRNA (bacteria) and ITS (fungi) gene regions. The biodegradability of these strains was evaluated by degradation experiments with cellulose and lignin as substrate. The results showed that the composition of the isolated microbial communities was different in each period, and microbial spatial distribution was dissimilar in the same period. In the recent 2020, the dominant bacterial genus was *Acinetobacter*, and the dominant fungal genera were *Penicillium*, *Aspergillus*, and *Cerrena*. *Acinetobacter* spp. can degrade cellulose and lignin. *Penicillium* spp., *Aspergillus* spp., and *Cerrena* spp. degraded cellulose but only *Cerrena* spp. could utilize lignin. These dominant strains may have a harmful effect on the Nanhai No. 1 Ancient Shipwreck. This study provides data on the airborne microbial community found inside the protective chamber where Nanhai No. 1 Shipereck is placed, which can be used as a reference basis for the future conservation of the ship.

## Introduction

The Nanhai No.1 Ancient Shipwreck is a wooden shipwreck of the Southern Song Dynasty, which was a merchant ship with an enormous hull. The Nanhai No. 1 is the most complete preserved shipwreck in China (Saiz-Jimenez and Gonzalez, 2007). The Nanhai No. 1 Ancient Shipwreck was discovered in 1987 within the limits of Taishan and Yangjiang in the Guangdong Province, and it was recovered from the sea in 2007. The ship was moved to the Maritime Silk Road Museum in the Guangdong province for archaeological excavation purposes (Liu, 2021). The archaeological works of the Nanhai No. 1 Ancient Shipwreck include whole salvage and the simultaneous exhibition and excavation in the museum (Geng, 2012). Visitors can see the archaeological excavations through the glass in exhibition hall. The hull of the wreck was well preserved, and the cargoes of the ship were stacked in an orderly manner. The cargoes included a large amount of fine porcelain, gold and silver utensils, and an assortment of household goods for the crew was recovered (Liu et al., 2018). The shipwreck is a physical material that reflects Chinese ancient shipbuilding techniques, navigation technology, the prosperous social life of the Southern Song Dynasty, and cultural exchanges between China and foreign countries (Liu, 2021). The Nanhai No. 1 Ancient Shipwreck has high research value as a typical representative of maritime wooden cultural relics.

Microorganisms from biological aerosols are similar to those on the surface of historical sites, indicating that microorganisms in the air may be one of the main sources of microorganisms on exposed cultural heritage sites (Li et al., 2022). The Nanhai No.1 wooden hull is made of pine and fir and it is currently exposed to the air, which is susceptible to erosion by biodeterioration-related microorganisms (Zhang et al., 2018). The microorganisms in the air can proliferate under suitable conditions of temperature, humidity, and light by using organic components in the materials (e.g., plant fibers and collagen) used in these cultural artifacts. They can either penetrate into the interior of the relics or secrete pigments and organic acids, affecting the structural features and artistic value of these artifacts (Nugari et al., 1993).

The atmosphere is the carrier of many bacteria and fungal spores (Duan et al., 2019). Some bacteria were reported to have the ability to degrade lignin, mainly including anaerobic species of *Bacillus*, *Acinetobacter*, *Flavobacterium*, *Micrococcus*, *Pseudomonas*, *Amphibacillus*. Among them, *Pseudomonas* spp. have the highest degradation activity (Tuomela et al., 2000; Bastian et al., 2009). Spores and mycelium fragments of fungi dispersed in the air can colonize the surface of artifacts. *Penicillium* spp. and *Cladosporium* spp. can secrete a variety of lignin and cellulose degrading enzymes and colonize wooden artifacts causing damage (Skora et al., 2015). Therefore, regular monitoring of airborne microorganisms at the historical sites and archaeological artifact’s location areas is necessary to assess the airborne microbial composition and the damage risks posed by these components (E et al., 2013).

The pollution by airborne microorganisms can affect the preservation of cultural heritage. Currently, there are no reports on the concentration, community structure, and distribution characteristics of airborne microorganisms in the protected environment of the Nanhai No.1 shipwreck. Therefore, this study monitored the airborne microbial community in the protected environment of the Nanhai No.1 shipwreck at the Maritime Silk Road Museum in Guangdong province. The concentration and community composition of culturable bacteria and fungi in the indoor and outdoor air environment of the museum were analyzed with culture-based and molecular identification techniques. The biodegradability potential of these dominant strains was additionally explored. The present study provides recommendations for the future conservation of shipwrecks, and it is important for early-warning microbial monitoring and preventive conservation of artifacts in the protective environment of wooden shipwrecks.

## Materials and methods

### Technical route

**Figure 1:**
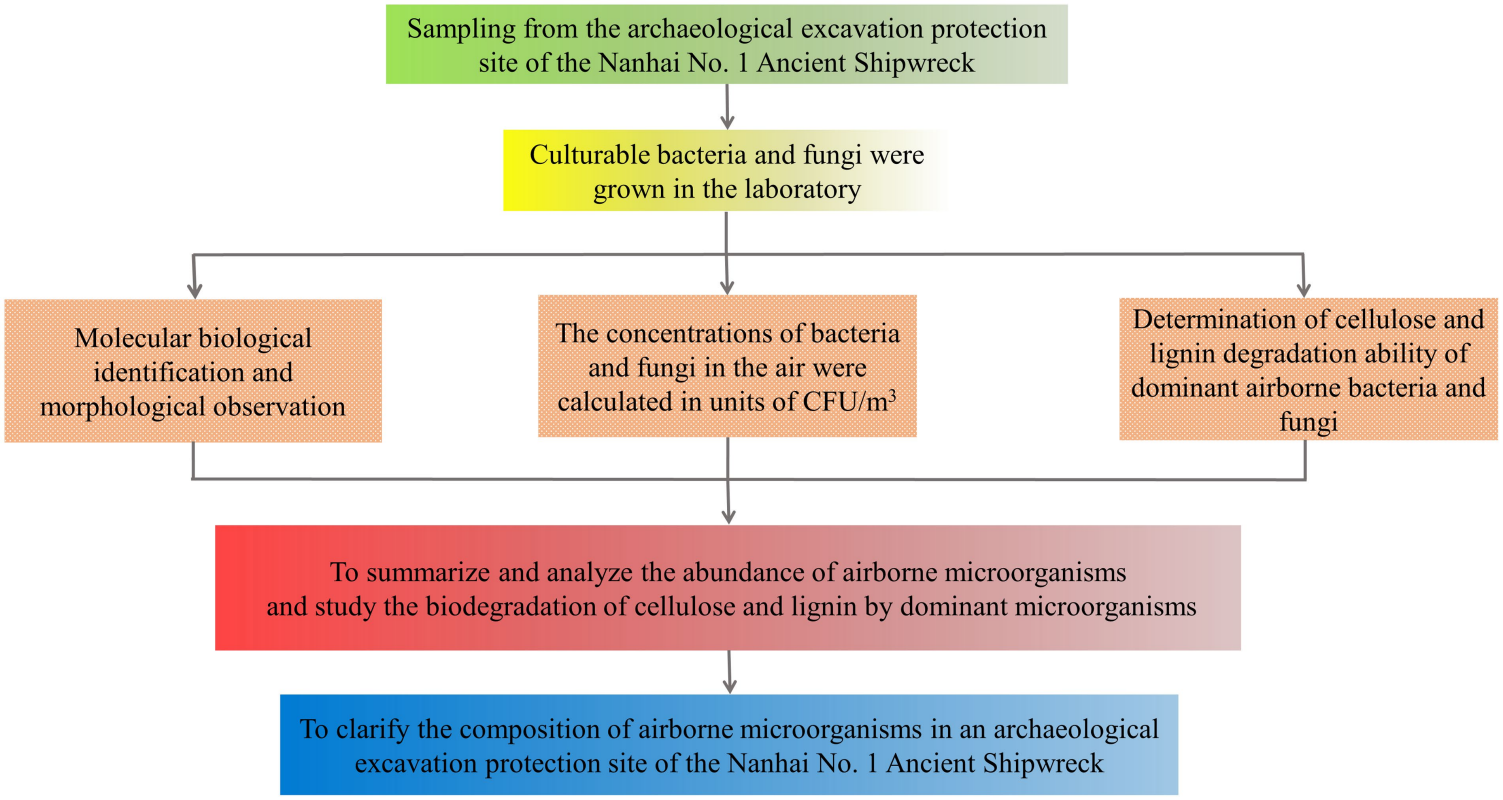
The flowchart of this research steps.

The research process of this study is shown in Figure 1.

### Sampling site

The ancient shipwreck of the Nanhai No. 1 is stored in the Maritime Silk Road Museum in Guangdong province, and its subsequent excavation and protection work was performed in a huge closed glass warehouse. The Maritime Silk Road Museum in Guangdong province is located on the west side of a long silver beach in the Hailing Island (Yangjiang City). The glass warehouse had an annual average temperature of 25.6°C and an annual average relative humidity of 84.1% (Liu et al., 2018), with a total area of approximately 8,738 square meters (Han et al., 2021). The remaining area of the hull is 22.95 m in length and 9.85 m in width (Han et al., 2021).

The airborne microbial composition where the shipwreck was located was sampled during five periods: April 2016, October 2016, June 2017, November 2019, and September 2020. The air was sampled from four directions (East, South, West, and North of the protective site), which were successively named as NHAP1-NHAP4.

**Figure 2:**
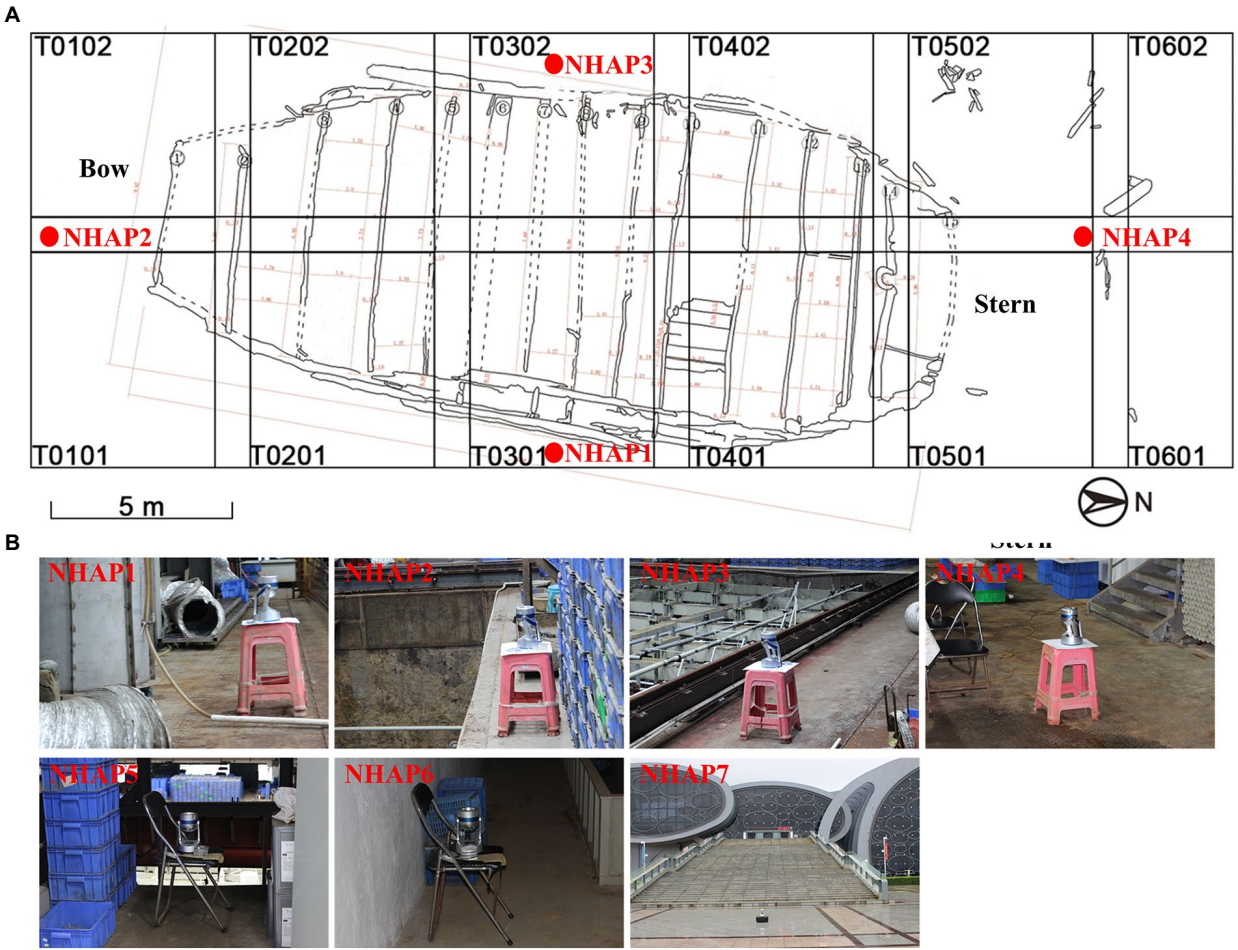
Specific locations of the air sampling points of the Nanhai No. 1 Ancient Shipwreck area. **(A)** T0101-T0602 is the archaeological exploration area on the wreck platform. **(B)** Images are samples collected at seven locations using air samplers.

Three additional sampling sites were added in September 2020. One site was the entrance of the glass warehouse (NHAP5), and it was located to the north of the archaeological probe of the wreck. Another site was the storeroom of the monitoring poll No. 4 (NHAP6), which was a dark area and had some of the debris (i.e., scattered timber) of the ship. Finally, the third site was outside the entrance of the Maritime Silk Road Museum in Guangdong province (NHAP7). The sampling locations of the Nanhai No. 1 Ancient Shipwreck are shown in Figure 2.

Meanwhile, we measured the temperature and humidity at the sampling sites with a TH101B hygrothermograph (Shengni). The range of temperature measurement was-30°C ~ 50°C, with error range is ≤ ± 1°C. The range of relative humidity measurement was 20% RH ~ 100% RH, with error range is ≤ ± 5% RH.

### Sampling protocol

We used Zr-2050 air sampler (Junray, China) for air sampling, which is an efficient single-stage porous impact sampler. This air sampler is based on the Anderson impact principle, and the impact velocity is 10.8 m/s, which can collect particles larger than 1 μm in the air. It draws air through a porous sampling head and impinges on a petri plate with a diameter of 90 mm. Microorganisms in the air are captured on the culture medium.

The LB (Luria-Bertani) media for airborne bacteria were composed of tryptone (10.0 g), yeast extract (5.0 g), NaCl (10.0 g), agar (20.0 g), and distilled water (1 l). The pH of the LB media were adjusted to 7.2 with 5 mol/l NaOH and autoclaved at 121°C for 20 min. The PDA (Potato Dextrose Agar) media for airborne fungi were composed of potato (200.0 g), glucose (20.0 g), agar (20.0 g), distilled water (1 l). The PDA media were autoclaved at 115°C for 20 min. The nutrient media used in this study were prepared in the laboratory according to the above formula. Air samples were collected at each location three times (One LB and one PDA petri dishes were exposed each time). The air flow rate was 100 l/min and the exposure time 2 min. We took the media back to the lab. LB media were incubated at 37°C for 2 days. PDA media were incubated at 28°C for 3 days. According to the color of colonies, size, shape, edge, luster, texture, and transparency, different strains were isolated in pure culture. Molecular identification of the purified culturable microorganisms was done by sequencing 16S rRNA for bacteria and ITS for fungi. Finally, the concentration of airborne microorganisms was calculated as colony forming units per cubic meter (CFU/m^3^) and a distribution map was drawn.

### DNA extraction and PCR amplification

The DNA extraction of airborne bacteria and fungi was performed using the hexadecyltrimethylammonium bromide (CTAB) method. The bacterial 16S rRNA genes were amplified using the 341F/907R primers [341F (5′- CCTACGGGAGGCAGCAG-3′) and 907R (5′- CCCCGTCAATTCATTTGAGTTT-3′)]. The fungal ITS regions were amplified using the ITS1/ITS4 primers [ITS1 (5′- TCCGTAGGTGAACCTGCGG-3′) and ITS4 (5′- CCTCCGCTTATTGATATGC-3′)]. The bacterial PCR mixtures had a total volume of 25 μl, containing 3 μl of genomic DNA, 2.5 μl of 10× reaction buffer, 2 μl of 2.5 mM deoxy-ribonucleoside triphosphate (dNTP) mix, 1 μl of 10 μm forward primer, 1 μl of 10 μm reverse primer, 0.5 μl of 5 U/μL TransTaq-T DNA polymerase (TransGen Biotech, Beijing, China), and 15 μl ddH_2_O. The fungal PCR mixtures had a total volume of 50 μl, including 2 μl of genomic DNA, 5 μl of 10× reaction buffer, 4 μl of 2.5 mM deoxy-ribonucleoside triphosphate (dNTP) mix, 2 μl of 10 μm forward primer, 2 μl of 10 μm reverse primer, 0.5 μl of 5 U/μL TransTaq-T DNA polymerase (TransGen Biotech, Beijing, China), and ddH_2_O to complete the 50 μl-final volume. The PCR products were detected by electrophoresis in 1% agarose gels.

The PCR products were sequenced by GENEWIZ (GENEWIZ, Beijing, China). The sequences obtained were analyzed using the National Center for Biotechnology Information (NCBI) BLAST program. Each isolate was compared against known taxa present in the GenBank database. We used blastn suite in the Nucleotide Blast.^1^ Algorithm parameters include general parameters (Max target sequences: 100, Short queries: select “Automatically adjust parameters for short input sequences,” Expect threshold: 0.05, Word size: 28, and Max matches in a query range: 0) and scoring parameters (Match/Mismatch Scores: 1, −2, and Gap Costs: Linear). All the obtained sequences were deposited in the NCBI GenBank under the accession numbers OP012703-OP012726 for bacteria and the accession numbers OP021989-OP022027 for fungi.

### Optical microscope observation

The morphology of the dominant fungi in the air was observed under a light microscope using the small chamber culture method. Using aseptic operation, small 1 × 1 cm pieces of solid PDA medium were taken with a sterile dissecting knife and placed on a slide in a sterilized Petri dish. These dominant strains were inoculated around the solid medium slice with an inoculating ring and further covered with a coverslip. Then, 1 ml of sterile glycerol was added dropwise on the filter paper in the sterilized Petri dish, the dish was covered with the lid. Samples were incubated at 28°C for 3 days. After that, the slides were removed and observed under a high magnification microscope.

An optical microscope (Nikon Eclipse E200, Japan) was used to observe the morphology of mycelia and spores under a 400 X-magnification. The scale bar on each micrograph is 10 μm.

### Determination of cellulose and lignin degradation capacity of dominant airborne microorganisms

Two Carboxyme thyl cellulose (CMC) agar media were prepared to assess the ability of the tested microorganisms to degrade cellulose. The CMC agar medium for bacteria was composed of CMC-Na (15.0 g), NaCl (5.0 g), KH_2_PO_4_ (1.0 g), MgSO_4_ (0.2 g), peptone (10.0 g), yeast extract (5.0 g), agar (18.0 g), and distilled water (1 l). Conversely, the CMC agar media for fungi had NaNO_3_ (2 g), K_2_HPO_4_ (1 g), MgSO_4_ (0.5 g), KCl (0.5 g), CMC (2 g), peptone (2 g), agar (17 g) and distilled water (1 l). All media were autoclaved at 121°C for 20 min.

The bacteria were inoculated on the middle of the plate and incubated at 37°C for 4 days. Also, 1 g/l Congo red solution was added to stain the plate, and the dye was discarded after 15 min. Then, 1 mol/l NaCl solution was added for dehydration purposes. After 15 min, the sodium chloride solution was removed, and the colony diameter and transparent circle diameter were measured (Ahmad et al., 2013). Fungal disks with a diameter of 7.5 mm were cut from the edge of actively growing strain colonies, and the fungal disks were transferred to CMC agar plates and cultured for 4 days at 28°C. A 5 ml iodine-potassium iodide solution (2.0 g potassium iodide, 1.0 g iodine, 300 ml double distilled water) was added and incubated for 5 min at room temperature in the dark to determine colony diameter and transparent circle diameter (Kasana et al., 2008). The cellulase activity of the tested microorganisms was initially determined based on the ratio of the hyaline circles (*H*) to the colony diameter (*D*). The larger the *H*/*D* value, the greater the capacity to degrade cellulose.

Additionally, four media were prepared to assess the lignin-degrading ability of the tested microorganisms. Three types of media for bacteria were prepared as follows: Medium I (Sodium lignosulfonate 2 g, (NH_4_)_2_SO_4_ 2 g, K_2_HPO_4_ 1 g, KH_2_PO_4_ 1.0 g, MgSO_4_ 0.2 g, CaCl_2_ 0.1 g, FeSO_4_ 0.05 g, MnSO_4_ 0.02 g, Agar 20 g, and distilled water 1 l; pH 7.0), Medium II (yeast extract 10 g, glucose 20 g, agar 20 g, aniline blue 0.1 g, and distilled water 1 l), Medium III (yeast extract 10 g, glucose 20 g, agar 20 g, ramazol brilliant blue 0.1 g, and distilled water 1 l). Medium I and II were autoclaved at 121°C for 20 min and medium III was autoclaved at 115°C for 20 min. For airborne fungi, the PDA medium included potato (200 g), glucose (20 g), agar (20 g), distilled water (1 l) supplemented with 0.04% guaiacol and autoclaved at 115°C for 20 min.

The bacteria were inoculated in Medium I and incubated at 37°C for 5 days for primary screening. The strains that could grow were then inoculated in medium II and medium III and incubated at 37°C for 4 days to determine the colony diameter and transparent circle diameter. The lignin activity of the tested strains was determined based on the ratio of the hyaline circles (*H*) to the colony diameter (*D*). The larger the *H*/*D* value, the greater the capacity to degrade lignin. Fungal disks with a diameter of 7.5 mm were cut from the edge of actively growing strain colonies. Then, the fungal disks were transferred to PDA agar plates and incubated at 28°C for 5 days and observed for color change. If there was a clear colorful circle, there was the ability to degrade lignin.

### Sample analysis

Airborne bacteria and fungi were counted as colony-forming units (CFU) on each plate by expressing these microorganisms in CFU units per cubic meter (CFU/m^3^) as shown in the following equation (Fang et al., 2005):


$$C=\frac{N\times 1000}{Q\times t}$$

where, *C* is the number of CFU/m^3^, *N* is the total number of a plate counts in CFU, *Q* is the air flow rate in L/min, and *t* is the sampling time in min.

All the experimental data were analyzed using Excel 2019 and SPSS Version 20.0 (SPSS, Standard Version).

## Results

### Identification of dominant airborne bacteria and fungi by culture-dependent methods

Twelve fungi were isolated in April 2016, three fungi were isolated in October 2016, five fungi were isolated in June 2017, five bacteria and nine fungi were isolated in November 2019, and nineteen bacteria and ten fungi were isolated in September 2020. Bacteria and fungi isolated on culture media were identified with molecular method. The 16S rRNA/ITS gene regions of the purified airborne bacteria/fungi were amplified and sequenced for analysis. The results are shown in Tables 1, 2.

**Table 1:** Molecular identification of bacteria isolated from the air in the protective environment of the Nanhai No. 1 Ancient Shipwreck.

Time	Bacteria	Closest related strain	Accession number	Query cover (%)	*E* values	Similarity (%)
November 2019	NH. A-B1	*Microbacterium*	MN463012.1	100	0.0	100
	NH. A-B2	*Corynebacterium*	MK415013.1	100	0.0	100
	NH. A-B3	*Escherichia coli*	MN732568.1	100	0.0	100
	NH. A-B4	*Paracoccus*	CP041042.1	100	0.0	100
	NH. A-B5	*Bacillus cereus*	MH732105.1	100	0.0	100
September 2020	NH. A-B6	*Enterobacter*	KY942132.1	100	0.0	100
	NH. A-B7	*Staphylococcus*	HQ154574.1	100	0.0	100
	NH. A-B8	*Bacillus*	MF276681.1	99	0.0	100
	NH. A-B9	*Acinetobacter*	MG719582.1	100	0.0	100
	NH. A-B10	*Chryseobacterium*	MT173808.1	100	0.0	100
	NH. A-B11	*Bacillus subtilis*	MT513998.1	99	0.0	100
	NH. A-B12	*Pseudomonas*	MT549181.1	99	0.0	100
	NH. A-B13	*Klebsiella*	EF433545.1	9%	0.0	99
	NH. A-B14	*Bacillus horikoshii*	MT534579.1	99	0.0	100
	NH. A-B15	*Lysinibacillus*	MH801072.1	100	0.0	99
	NH. A-B16	*Pantoea*	MT071155.1	100	0.0	100
	NH. A-B17	*Citrobacter*	KX214776.1	100	0.0	99
	NH. A-B18	*Serratia marcescens*	CP031316.1	99	0.0	100
	NH. A-B19	*Corynebacterium*	MN784239.1	99	0.0	100
	NH. A-B20	*Micrococcus*	MH731924.1	99	0.0	100
	NH. A-B21	*Moraxella osloensis*	MT328624.1	99	4e-72	99
	NH. A-B22	*Microbacterium*	MH537665.1	99	0.0	99
	NH. A-B23	*Paracoccus*	CP041042.1	100	0.0	95
	NH. A-B24	*Kocuria*	MN904945.1	99	0.0	100

**Table 2:** Molecular identification of fungi isolated from the air in the protective environment of the Nanhai No. 1 Ancient Shipwreck.

Time	Fungi	Closest related strain	Accession number	Query cover (%)	*E* values	Similarity (%)
April 2016	NH. A-20	*Aspergillus versicolor*	KP027423.1	97	0.0	99
	NH. A-21	*Psathyrella candolleana*	AF345810.1	89	0.0	99
	NH. A-22	*Diaporthe*	MF618354.1	93	1e-165	99
	NH. A-23	*Chaetomium globosum*	KU293593.1	93	0.0	99
	NH. A-24	*Cladosporium sphaerospermum*	KT962859.1	84	1e-176	99
	NH. A-25	*Coprinellus radians*	MW081259.1	90	0.0	99
	NH. A-26	*Aspergillus*	JQ717354.1	97	0.0	99
	NH. A-27	*Peniophora*	JF925333.1	93	0.0	99
	NH. A-28	*Basidiomycota*	KF990156.1	93	0.0	99
	NH. A-29	*Ceriporia*	KR155097.1	92	0.0	99
	NH. A-30	*Fusarium*	MH681596.1	93	0.0	99
	NH. A-31	*Phlebia*	KJ605162.1	94	0.0	99
October 2016	NH. A-32	*Penicillium oxalicum*	JN542545.1	99	0.0	99
	NH. A-33	*Aspergillus aculeatus*	KM520043.1	99	0.0	99
	NH. A-34	*Rhizopus arrhizus*	MT603964.1	94	0.0	99
June 2017	NH. A-35	*Penicillium*	MT498093.1	98	0.0	98
	NH. A-36	*Neurospora*	KP027411.1	98	0.0	99
	NH. A-37	*Neurospora sitophila*	KM588213.1	97	0.0	99
	NH. A-38	*Aspergillus aculeatus*	KC621081.1	99	0.0	99
	NH. A-39	*Neurospora intermedia*	KT844676.1	99	0.0	99
November 2019	NH. A-1	*Penicillium*	MN518406.1	100	0.0	100
	NH. A-2	*Cladosporium*	MH985344.1	100	0.0	100
	NH. A-3	*Penicillium citrinum*	MN736554.1	100	1e-169	100
	NH. A-4	*Talaromyces*	MH935987.1	100	0.0	100
	NH. A-5	*Cladosporium*	KY621330.1	99	0.0	99
	NH. A-6	*Penicillium*	MN640089.1	99	0.0	100
	NH. A-7	*Penicillium pimiteouiense*	KC344973.1	100	0.0	100
	NH. A-8	*Penicillium sclerotiorum*	MG827186.1	100	0.0	100
	NH. A-9	*Talaromyces pinophilus*	MF686811.1	100	0.0	100
September 2020	NH. A-10	*Penicillium*	JN687974.1	97	0.0	99
	NH. A-11	*Aspergillus*	MK431431.1	99	0.0	100
	NH. A-12	*Cerrena*	KX013197.1	100	0.0	100
	NH. A-13	*Talaromyces*	MT530095.1	100	0.0	100
	NH. A-14	*Cladosporium*	MH725789.1	99	0.0	99
	NH. A-15	*Trichoderma*	MK870388.1	99	0.0	100
	NH. A-16	*Colletotrichum*	MT492128.1	99	0.0	100
	NH. A-17	*Scopulariopsis*	EU821474.1	100	3e-167	94
	NH. A-18	*Castanediella*	MN660236.1	93	0.0	95
	NH. A-19	*Neofusicoccum parvum*	MH183388.1	100	0.0	99

### Number and composition of microorganisms in the air of the Nanhai No. 1 Ancient Shipwreck in 2020

Samples collected in September 2020 were separated, purified, identified, classified, and grouped with the initial plate counts. The concentration of airborne bacteria and fungi was different at different sampling sites. The concentration of airborne bacteria on the east side of the protection site (NHAP1) was 2,680 CFU/m^3^ (Table 3). It was the most abundant and had a significant difference compared to the other sites (*p* < 0.05). The fungal abundance at the outside entrance to Maritime Silk Road Museum in Guangdong province was relatively high (NHAP7), which was 195 CFU/m^3^ (Table 3), showing a significant difference with other sites (*p* < 0.05). In the other sites, fungal abundance was more even.

**Table 3:** Total bacterial and fungal concentrations, temperature, and humidity in the air at each sampling point in 2020.

Sampling point	Temperature (°C)	Humidity (%)	Total bacterial (CFU/m^3^)	Total fungi (CFU/m^3^)
NHAP1	24.5	83	2,680 ± 15^a^	92.5 ± 22.5^c^
NHAP2	24.5	83	55 ± 5^d^	120 ± 5^c^
NHAP3	24.5	83	145 ± 5^c^	112.5 ± 12.5^c^
NHAP4	24.5	83	570 ± 145^b^	165 ± 15^b^
NHAP5	24.5	83	37.5 ± 2.5^e^	112.5 ± 12.5^c^
NHAP6	24.5	83	162.5 ± 52.5^c^	137.5 ± 22.5^bc^
NHAP7	24.5	76	25 ± 15^f^	195 ± 5^a^

Different letters denote significant differences (*P* < 0.05).

**Figure 3:**
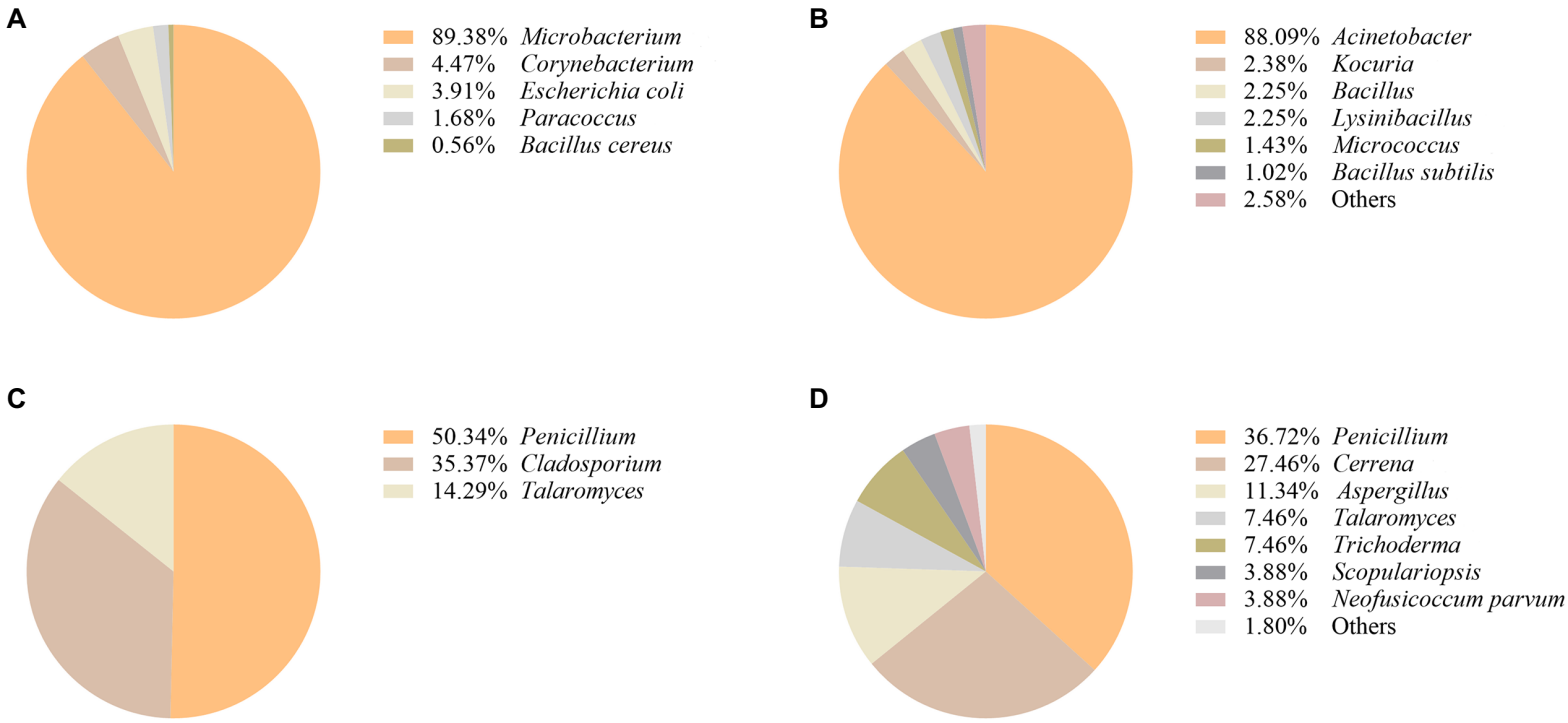
Composition of airborne bacterial communities in the protected environment of Nanhai No.1 shipwreck in 2019 **(A)** and 2020 **(B)**. Composition of airborne fungal communities in the protected environment of Nanhai No.1 shipwreck in 2019 **(C)**, and 2020 **(D)**.

It was found that the most represented airborne bacteria changed from *Microbacterium* to *Acinetobacter* during the 2019–2020 period (Figures 3A,B). *Acinetobacter* accounted for 88.09% of the total number of airborne bacteria (Figure 3B). Regarding the isolated fungal community (Figures 3C,D), it was found that *Penicillium* accounted for the largest percentage of airborne fungi in both years (50.34 and 36.72% of the total fungi in 2019 and 2020, respectively). The second most abundant fungal genus was *Cerrena* representing 27.46% of the total number of airborne fungi in 2020, followed by *Aspergillus* with 11.34% (Figure 3D).

**Figure 4:**
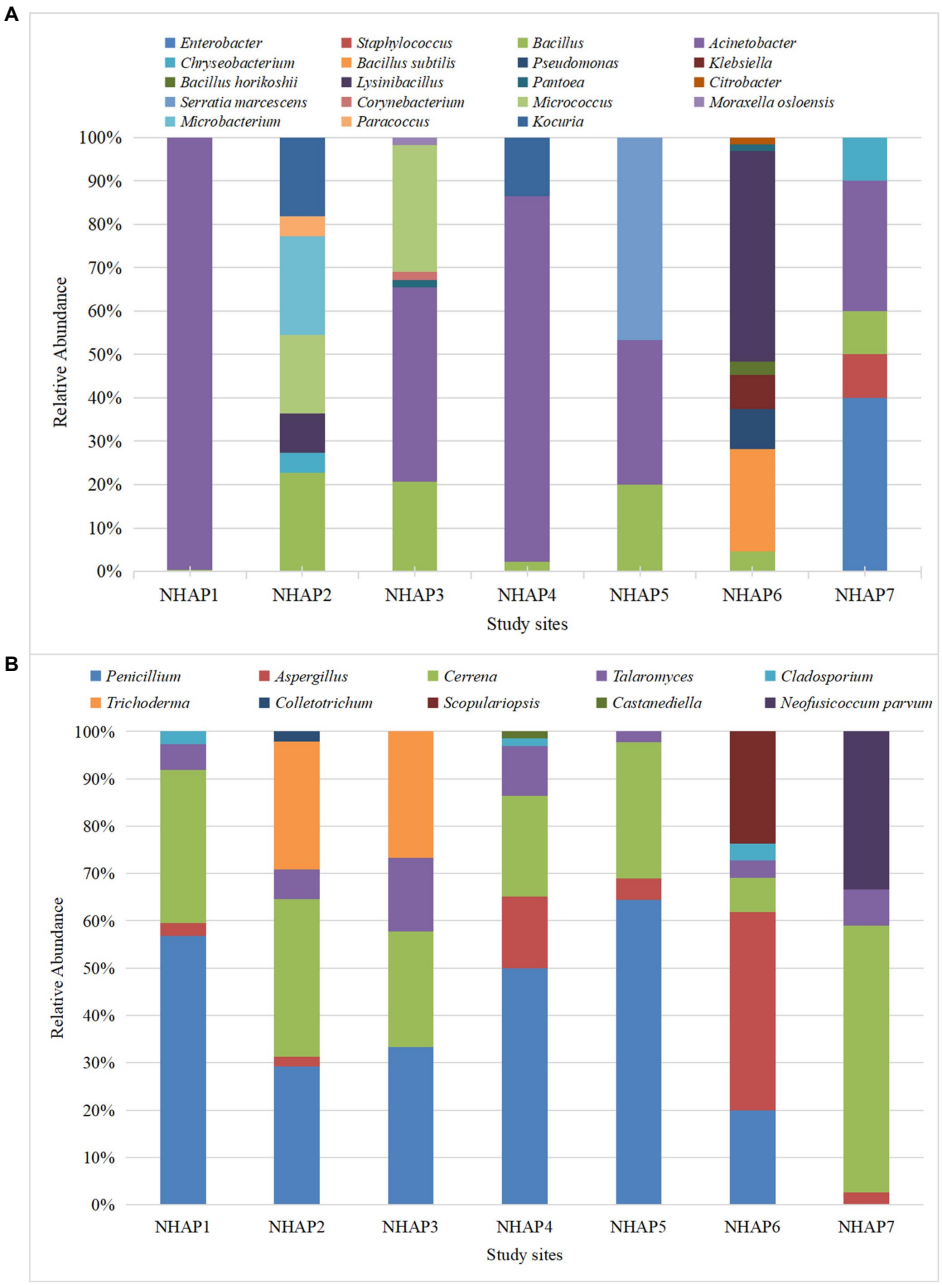
Relative abundance of airborne bacteria **(A)** and fungi **(B)** at each of the Nanhai No.1 shipwreck sampling sites in 2020.

Figure 4A shows the relative abundance of airborne bacteria at each site. The most abundant airborne bacteria in the NHAP1 were *Acinetobacter.* NHAP2, NHAP3 and NHAP4 had mostly *Bacillus* and *Acinetobacter. Serratia marcescens* was only found at NHAP5. *Klebsiella* was only found at NHAP6. *Enterobacter* and *Staphylococcus* were only found at NHAP7.

Regarding the fungi, NHAP1-NHAP5 had *Penicillium*, *Cerrena*, and *Talaromyces.* NHAP6 accounted for most of *Aspergillus*, and NHAP7 accounted for most of *Cerrena*. *Scopulariopsis* was only found in NHAP6 and *Neofusicoccum parvum* was only found at NHAP7 (Figure 4B).

### Determination of cellulose and lignin degradation ability of dominant airborne bacterium

**Figure 5:**
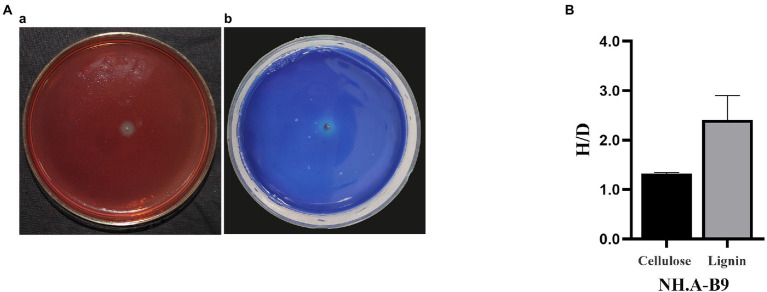
Degradation of cellulose and lignin by *Acinetobacter* (NH. A-B9). **(A)** Degradation images of NH. A-B9. (a) NH. A-B9 degraded cellulose. (b) NH. A-B9 degraded lignin. **(B)** The cellulose hydrolase activity and laccase production capacity of NH. A-B9 were initially determined based on the ratio of H and D (H/D). The error bars represented the standard deviation.

*Acinetobacter* (NH. A-B9) was the dominant airborne bacterium, and the ability to degrade cellulose and lignin was tested to assess its potential effect on hull conservation (Figure 5). NH. A-B9 was able to degrade cellulose and lignin to produce degradation circles. NH. A-B9 had weak ability to degrade cellulose (H/D < 2) and strong ability to degrade lignin (H/D > 2).

### Determination of cellulose and lignin degradation ability of dominant airborne fungi

**Figure 6:**
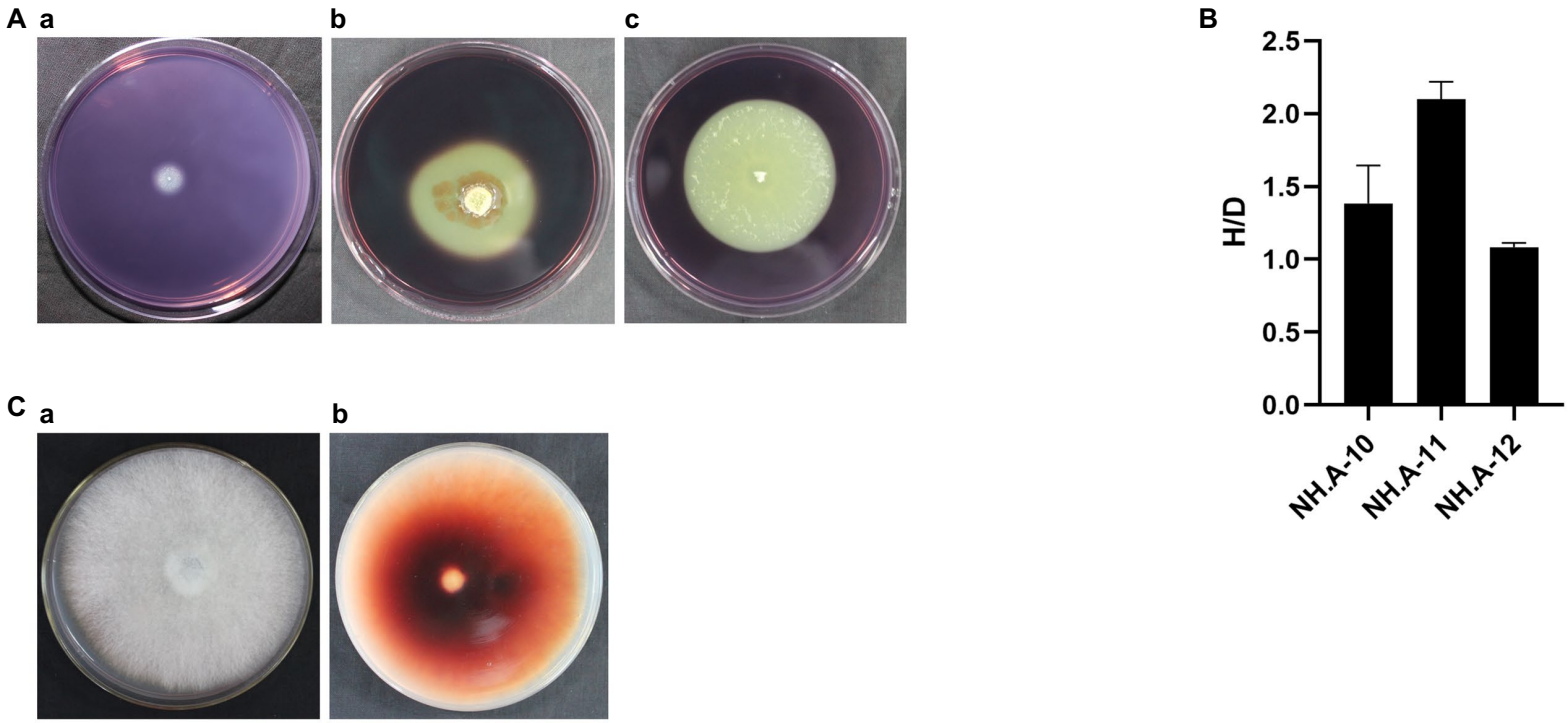
Ability of the dominant fungi to degrade cellulose and lignin. **(A)** Degradation images of dominant fungi. (a) *Penicillium* (NH. A-10). (b) *Aspergillus* (NH. A-11). (c) *Cerrena* (NH. A-12). **(B)** The preliminary determination of cellulose hydrolase activity was assessed for these three fungi based on the ratio of H and D (H/D). The error bars represented the standard deviation. **(C)** NH. A-12 grown on the PDA-guaiacol medium at 28°C for 5 days showing (a) the front and (b) back side of the medium.

The degradation ability of cellulose and lignin by the most abundant airborne fungi detected in this study, *Penicillium* (NH. A-10), *Aspergillus* (NH. A-11), and *Cerrena* (NH. A-12), was assessed to investigate the possible effect of airborne fungi on hull protection. These genera were able to degrade cellulose (see degradation loops in Figure 6A). The biodegradation ability, from the highest to the lowest, was NH. A-11 (H/D > 2), NH. A-10 and NH. A-12 (H/D < 2) (Figure 6B). Only NH. A-12 was found to have brownish-red areas (Figure 6C) which implies that can use lignin as a substrate.

### Micromorphological observation of dominant airborne fungal isolates at the protected site of the Nanhai No.1 Shipwreck in 2020

**Figure 7:**
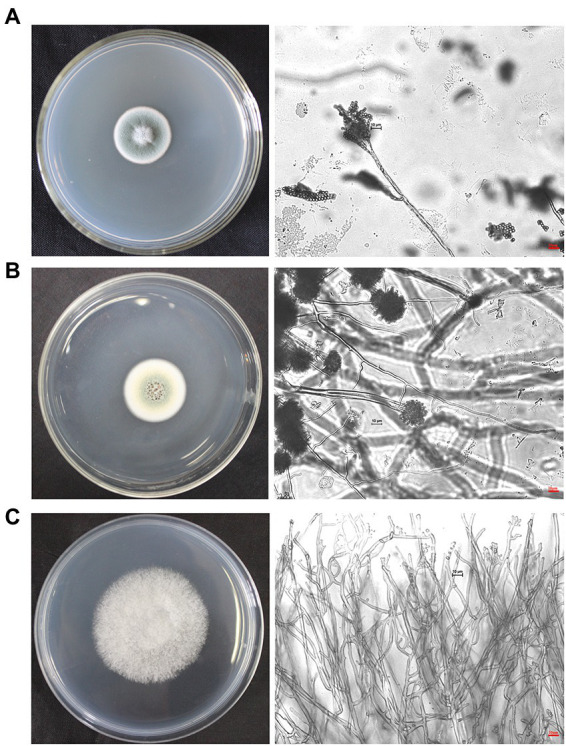
Colonies and micromorphological characteristics of three dominant fungi isolated from September 2020 samples (×400). The scale bars of the microphotographs were 10 μm. **(A)** *Penicillium.* **(B)** *Aspergillus*. **(C)** *Cerrena*.

The dominant airborne fungi isolated in September 2020 were *Penicillium*, *Aspergillus* and *Cerrena*. Colony and micromorphological characteristics of these three fungi were observed and recorded with optical microscope (Figure 7). The conidiophores of *Penicillium* were penicillus arrangement without vesicle, and they produced many phialides. There are biverticillate or terverticillate penicillus. Spheroidal or ovoid conidia were on the phialides (Shao and Shen, 1984, p. 75). Vesicle is the swelling in *Aspergillus* conidiogenous cells. There are metulas and phialides on the vesicle. Phialides produce spheroidal conidia in long chains (Zhou, 1993, p. 60). The colony of *Cerrena* was white and villous. It has three types of hyphae, namely reproductive hyphae, skeletal hyphae and twining hyphae. The reproductive hypha has abundant branches and intervals. The skeleton mycelium has thick wall, no septum and no branching. Twining hyphae with multiple branches, twining other hyphae (Alexopoulos et al., 1983, p. 386). The characteristics of the three fungi are consistent with our observations.

## Discussion

This study assessed the composition of culturable airborne microbial colonies in the protective environment of the Nanhai No. 1 Ancient Shipwreck excavation. There were *Acinetobacter*, *Bacillus*, *Pseudomonas*, *Aspergillus*, *Cladosporium*, and *Penicillium*. These genera are common taxa in many cultural heritage sites. The dominant airborne microorganisms of the China Three Gorges Museum are *Acinetobacter*, *Aspergillus* and *Penicillium* (Tang et al., 2014). *Penicillium* and *Cladosporium* have been reported in murals and caves (Gorbushina et al., 2004; Jurado et al., 2009). The main airborne bacteria in Mogao Grottoes of Dunhuang are *Bacillus*, *Micrococcus*, and *Pseudomonas*, and the airborne bacteria found in the Maijishan Grottoes are mainly *Bacillus* (Wang et al., 2010; Duan et al., 2019). The dominant airborne fungi found in the Tokyo Art Museum are *Cladosporium* and *Aspergillus* (Abe, 2010). In the National Archive of the Republic of Cuba, the airborne fungi were mainly *Aspergillus*, *Cladosporium* and *Penicillium* (Borrego et al., 2022). The dominant airborne fungi of the National Maritime Museum of China are *Penicillium* and *Cladosporium* (Zhang et al., 2019).

Airborne microorganisms were sampled and studied for 4 years (2016–2017, 2019–2020). This study mainly focused on culturable airborne fungi during the years 2016 and 2017. Fungi have a relatively high potential for biodeterioration (Bastian et al., 2010). The hull of the Nanhai No. 1 has some parts contaminated by fungi, and the long-term biodegradation of destructive fungi can lead to the destruction of the wooden structure of the hull. Some bacteria such as *Bacillus* and *Pseudomonas* (Paliwal et al., 2015; Takeuchi et al., 2017) can also degrade cellulose and lignin and destroy the hull structure. Therefore, bacteria were added as a new indicator in the 2019–2020 period.

There were more types of culturable airborne bacteria and fungi at each sampling site in September 2020 than in November 2019. This finding is linked to seasonal changes (Tanaka et al., 2015). In the same season, the concentrations of airborne bacteria and fungi in the air were different from each site, which may be related to microenvironmental conditions, human activity, ventilation systems, different substrata, etc. (Tanaka et al., 2015; Lu et al., 2022).

In 2020, the distribution of culturable airborne fungi in protected sites was more uniform. The highest concentration of airborne bacteria was found on the east side of the conservation site (NHAP1), probably due to the connection to the ventilation system, followed by a higher number on the north side of the site (NHAP4), probably due to staff activities. It has been reported that atmospheric bacteria concentrations are positively correlated with ground disturbances such as those caused by human activities (Tang et al., 2016). Moreover, these activities can stir up dust and small soil particles on the ground. *Acinetobacter* was the most abundant airborne bacterium in the eastern side of the excavation protection site. *Acinetobacter* is widely distributed in the environment, mainly in water bodies and soil, and easily survives in moist environments. This genus is very strong in adhesion (Su et al., 2020). *Acinetobacter* may have adhered to the ventilation ducts, causing the largest abundance percentage on the east side of the site. It was found that the fungal concentration was much less than the bacterial concentration on the east side of the protection site (NHAP1), probably due to the ventilation effect of the machine.

The concentration of airborne fungi in the storage room of monitoring pool No. 4 (NHAP6) did not differ much from the protection site, and the concentration of airborne bacteria was at a medium level. The monitoring poll No.4 was in a dark environment with low personnel movement. The concentration of culturable airborne fungi was the highest outside the entrance of the Maritime Silk Road Museum in Guangdong province (NHAP7). It rained while sampling at NHAP7. After rainfall certain fungi increase in the air. Rainfall has a washing out effect on bioaerosols, but rain contributes in spore release (Zhou and Xing, 1986, p. 97). It might explain why the concentration of fungi at the NHAP7 was higher than that in the indoor environment. We also found that the concentration of airborne bacteria was much lower than that of airborne fungi at the outside entrance of Maritime Silk Road Museum in Guangdong province.

It is worth noting that there are some strains that are only present at a certain sampling site. *S. marcescens* was only found at the entrance of the protective site (NHAP5). *Klebsiella* and *Scopulariopsis* were only found at the storeroom of monitoring poll No.4 (NHAP6). *Enterobacter*, *Staphylococcus* and *N. parvum* were only found at the outside entrance at the museum (NHAP7). NHAP5 is the entrance, and air circulation and staff traffic are more frequent than the protective site. The presence of these genera can be attributed to the staff entering the storeroom of monitoring pool No. 4 (NHAP6). The museum is located by the sea, facing South China Sea. The outside entrance of the museum is wide, with some green plants planted. The location may be the reason for the existence of these strains.

We explored the biodegradability of dominant airborne microorganisms with cellulose and lignin as substrates to reflect their potential harm to hull. *Acinetobacter* spp. and *Cerrena* spp. may cause irreversible damage to the hull by degrading cellulose and lignin of the ship wood. *Penicillium* spp. and *Aspergillus* spp. mainly degrade cellulose. These dominant strains have some ability to biodegrade wood and may have a harmful effect on the Nanhai No. 1 Ancient Shipwreck.

## Conclusion

The current archaeological excavation work of the Nanhai No. 1 Ancient Shipwreck has been finished. The hull was exposed to air for a long time, and the high relatively humidity provided an environment conducive to the growth of microorganisms. On the one hand, these microorganisms can damage the aesthetics of cultural heritage. On the other hand, they damage the structure of cultural heritage. Therefore, airborne microorganisms are considered as a potential hazard for the conservation of the Nanhai No.1 shipwreck.

In summary, the composition of culturable microorganisms in the air of the protected environment of the Nanhai No.1 shipwreck changed from year to year. Besides time, these changes of culturable microbial composition can be related to microenvironmental conditions, seasons, meteorological parameters, and human activities. This study concludes that the shipwreck of the Nanhai No.1 is at greater risk of microbial erosion. The airborne microorganisms in the protective site should be monitored for a long time to provide scientific basis for preventive protection.

In the process of protection, it is necessary to pay attention to some influencing factors, such as the disturbance of air by staff activities, cleaning of the ventilation system, etc. The Nanhai No.1 can be better preserved by taking some measures. For instance, the staffs, prior to entering the heritage site, must process proper disinfection and be equipped with protective gears. Also, the installation of microbial filtration devices and the conduction of regular disinfection are highly necessary.

## Data availability statement

The datasets presented in this study can be found in online repositories. The names of the repository/repositories and accession number (s) can be found in the article/supplementary material.

## Author contributions

JP: conceived and planned this study and reviewed and edited the manuscript. KM, YW, YH, and XH: performed the experiments. JD and YC: provided assistance during the experiments. KM, XY, CW, and PG: analyzed the data. KM: wrote the manuscript. All authors have read and agreed in the published version of the manuscript.

## Funding

This study was funded by the Natural Science Foundation of Tianjin (19JCZDJC33700) and the National Key Research and Development Program of China (2020YFC1521800).

## Conflict of interest

The authors declare that the research was conducted in the absence of any commercial or financial relationships that could be construed as a potential conflict of interest.

## Publisher’s note

All claims expressed in this article are solely those of the authors and do not necessarily represent those of their affiliated organizations, or those of the publisher, the editors and the reviewers. Any product that may be evaluated in this article, or claim that may be made by its manufacturer, is not guaranteed or endorsed by the publisher.

## Footnotes

^1^ https://blast.ncbi.nlm.nih.gov/Blast.cgi

## References

[ref1] Abe K. (2010). Assessment of the environmental conditions in a museum storehouse by use of a fungal index. Int. Biodeterior. Biodegradation 64, 32–40. doi: 10.1016/j.ibiod.2009.10.004

[ref2] Ahmad B. Nigar S. Shah S. S. Bashir S. Ali J. Yousaf S.. (2013). Isolation and identification of cellulose degrading bacteria from municipal waste and their screening for potential antimicrobial activity. World Appl. Sci. J. 27, 1420–1426. doi: 10.5829/idosi.wasj.2013.27.11.81162

[ref3] Alexopoulos C. J. Mims C. W. Blackwell M. (1983). *Introduction to Funcology*. Translated from the Chinese by Y. Yu. Beijing: Agricultural Press.

[ref4] Bastian F. Alabouvette C. Jurado V. Saiz-Jimenez C. (2009). Impact of biocide treatments on the bacterial communities of the Lascaux Cave. Naturwissenschaften 96, 863–868. doi: 10.1007/s00114-009-0540-y, PMID: 19404600

[ref5] Bastian F. Jurado V. Nováková A. Alabouvette C. Saiz-Jimenez C. (2010). The microbiology of Lascaux Cave. Microbiolog 156, 644–652. doi: 10.1099/mic.0.036160-0, PMID: 20056706

[ref6] Borrego S. Vivar I. Molina A. (2022). Air-and dustborne fungi in repositories of the National Archive of the Republic of Cuba. Microbial Cell. 9, 103–122. doi: 10.15698/mic2022.05.776, PMID: 35647176 PMC9113668

[ref7] Duan Y. L. Wu F. S. Wang W. F. He D. P. Ma Q. Dong G. Q. (2019). Spatial and temporal distribution characteristics of the airborne bacteria in the Maijishan Grottoes, China. Acta Microbiol. Sin. 59, 45–156. doi: 10.13343/j.cnki.wsxb.20180094

[ref8] E J. Wu F. S. Wang W. F. Chen G. L. Zhao L. Y. He D. P.. (2013). Monitoring and research on microbes in the environment of the wall paintings in No. 5 of the Wei and Jin Tombs. Dunhuang Res. 6, 109–116. doi: 10.13584/j.cnki.issn1000-4106.2013.06.016

[ref9] Fang Z. Ouyang Z. Hua Z. Wang X. Hu L. (2005). Culturable airborne fungi in outdoor environments in Beijing, China. Sci. Total Environ. 350, 47–58. doi: 10.1016/j.scitotenv.2005.01.032, PMID: 16227072

[ref10] Geng M. (2012). The “Nanhai No. 1” open protection and repair laboratory construction of excavated cultural relics. Sci. Technol. Innov. Herald. 4, 228–230. doi: 10.16660/j.cnki.1674-098x.2012.04.127

[ref11] Gorbushina A. A. Heyrman J. Dornieden T. Gonzalez-Delvalle M. Krumbein W. E. Laiz L.. (2004). Bacterial and fungal diversity and biodeterioration problems in mural painting environments of St. Martins church (Greene-Kreiensen Germany). Int. Biodeterior. Biodegrad. 53, 13–24. doi: 10.1016/j.ibiod.2003.07.003

[ref12] Han Y. Q. Huang X. D. Wang Y. Du J. Ma K. X. Chen Y.. (2021). Fungal community and biodeterioration analysis of hull wood and its storage environment of the Nahai No. 1 Shipwreck. Front. Microbiol. 11:609475. doi: 10.3389/FMICB.2020.609475 33519760 PMC7843524

[ref13] Jurado V. Fernandez-Cortes A. Cuezva S. Laiz L. Cañaveras C. J. SanchezMoral S.. (2009). The fungal colonization of rock-art caves: experimental evidence. Naturwissenschaften 96, 1027–1034. doi: 10.1007/s00114-009-0561-6, PMID: 19484211

[ref14] Kasana R. Salwan R. Dhar H. Dutt S. Gulati A. (2008). A rapid and easy method for the detection of microbial cellulases on agar plates using gram’s iodine. Current Microbiol. 57, 503–507. doi: 10.1007/s00284-008-9276-8, PMID: 18810533

[ref15] Li T. Cai Y. Ma Q. (2022). Microbial diversity on the surface of historical monuments in Lingyan Temple, Jinan, China. Microb. Ecol. 83, 1–11. doi: 10.1007/s00248-021-01955-w 34997309

[ref16] Liu D. M. (2021). Analysis on the exhibition “The sea route: Nanhai I shiwreck and maritime trade in the southern song dynasty”. J. Arts Folklore. 4, 4–9.

[ref17] Liu Z. Fu T. Hu C. Shen D. Macchioni N. Sozzi L.. (2018). Microbial community analysis and biodeterioration of waterlogged archaeological wood from the Nanhai No. 1 Shipwreck during storage. Sci. Rep. 8, 7170. doi: 10.1038/s41598-018-25484-8, PMID: 29740020 PMC5940862

[ref18] Lu Y. Wang X. Almeida L. C. S. D. S. Pecoraro L. (2022). Environmental factors affecting diversity, structure, and temporal variation of airborne fungal communities in a research and teaching building of Tianjin University, China. J. Fungi 8, 431. doi: 10.3390/jof8050431, PMID: 35628687 PMC9144611

[ref19] Nugari M. P. Realini M. Roccardi A. (1993). Contamination of mural paintings by indoor airborne fungal spores. Aerobiologia 9, 131–139. doi: 10.1007/BF02066254

[ref20] Paliwal R. Uniyal S. Rai J. P. N. (2015). Evaluating the potential of immobilized bacterial consortium for black liquor biodegradation. Environ. Sci. Pollut. Res. 22, 6842–6853. doi: 10.1007/s11356-014-3872-x, PMID: 25433900

[ref21] Saiz-Jimenez C. Gonzalez J. (2007). Aerobiology and cultural heritage: some reflections and future challenges. Aerobiologia 23, 89–90. doi: 10.1007/s10453-007-9059-x

[ref22] Shao L. P. Shen R. X. (1984). Fungal Taxonomy. Beijing: China Forestry Press.

[ref23] Skora J. Gutarowska B. Pielech-Przybylska K. Stepien L. Pietrzak K. (2015). Assessment of microbiological contamination in the work environments of museums, archives and libraries. Aerobiologia 31, 389–401. doi: 10.1007/s10453-015-9372-8, PMID: 26346115 PMC4556743

[ref24] Su J. F. Zhang H. Huang T. L. Hu X. F. Chen C. L. Liu J. R. (2020). The performance and mechanism of simultaneous removal of fluoride, calcium, and nitrate by calcium precipitating strain *Acinetobacter* sp. H12. Ecotoxicol. Environ. Saf. 187:109855. doi: 10.1016/j.ecoenv.2019.109855, PMID: 31689622

[ref25] Takeuchi Y. Khawdas W. Aso Y. Ohara H. (2017). Microbial fuel cells using *Cellulomonas* spp. with celluioseas fuel. J. Biosci. Bioeng. 123, 358–363. doi: 10.1016/j.jbiosc.2016.10.009, PMID: 27818074

[ref26] Tanaka D. Terada Y. Nakashima T. Sakatoku A. Nakamura S. (2015). Seasonal variations in airborne bacterial community structures at a suburban site of Central Japan over a 1-year time period using PCR-DGGE method. Aerobiologia 31, 143–157. doi: 10.1007/s10453-014-9353-3

[ref27] Tang H. Fan W. Q. Zhou L. K. Wang C. (2014). Airbome microbial content in the temporary exhibition halls of China three gorges museum. Chin. J. Microecol. 26, 420–424. doi: 10.13381/j.cnki.cjm.201404012

[ref100] Tang H. Zhou L. K. Wang C. Zhang L. L. (2016). Richness and diversity of airmicroorganisms during peak period of visitors flow in museum exhibition hall. J. Environ. Health. 33, 707–711. doi: 10.16241/j.cnki.1001-5914.2016.08.013, PMID: 32372375

[ref28] Tuomela M. Vikmanb M. Hatakka A. (2000). Biodegradation of Iignin in a compost environment. Bioresour. Technol. 72, 169–183. doi: 10.1016/S0960-8524(99)00104-2

[ref29] Wang W. F. Ma Y. T. Ma X. An L. Feng H. (2010). Seasonal variations of airborne bacteria in the Mogao Grottoes, Dunhuang, China. Int. Biodeterior. Biodegr. 64, 309–315. doi: 10.1016/j.ibiod.2010.03.004

[ref30] Zhang F. Li L. Sun M. Hu C. T. Zhang Z. G. Liu Z. J.. (2019). Fungal community analyses of a pirogue from the tang dynasty in the national maritime museum of China. Appl. Sci. 9, 4129. doi: 10.3390/app9194129

[ref31] Zhang Z. G. Sun J. Xi G. l. Li N. S. Shen D. W. (2018). Identification of timber species and analysis of timber used for the hull of Nanhai No. 1. Underwater Archaeol. 4, 197–206.

[ref32] Zhou D. Q. (1993). Microbiology Tutorial. Beijing: Higher Education Press.

[ref33] Zhou Y. L. Xing L. J. (1986). Mycology. Beijing: Higher Education Press.

